# Growing burden of diabetes in Pakistan and the possible role of arsenic and pesticides

**DOI:** 10.1186/s40200-014-0117-y

**Published:** 2014-12-14

**Authors:** Haji Bahadar, Sara Mostafalou, Mohammad Abdollahi

**Affiliations:** Department of Toxicology and Pharmacology, Faculty of Pharmacy and Pharmaceutical Sciences Research Center, International Campus, Tehran University of Medical Sciences, Tehran, Iran; Department of Pharmacology and Toxicology, School of Pharmacy, Ardebil University of Medical Sciences, Ardebil, Iran

**Keywords:** Arsenic, Diabetes mellitus, Environmental pollutants, Heavy metals, Pakistan, Pesticides, Review

## Abstract

This review is undertaken to address the possible role of arsenic and pesticides in the prevalence of diabetes in Pakistan and to highlight a resourceful targeted research in this area.

A bibliographic search of scientific databases was conducted with key words of “epidemics of diabetes in Pakistan”, “diabetes in Asia”, “diabetes mellitus and environmental pollutants”, “diabetes mellitus and heavy metals”, “diabetes mellitus and pesticides”, “prevalence of pesticides in Pakistan”, and “heavy metals contamination of drinking water, “vegetables and fruits in Pakistan”. More than 200 articles were examined. Studies reporting the prevalence of diabetes mellitus (DM), pesticides and heavy metal contamination of drinking water, fruits and vegetables were included in the study. According to WHO 2011 report, about 12.9 million people are suffering from DM and the number is constantly increasing. Water pollution is a major public health threat in Pakistan. Most of the people in Pakistan are exposed to arsenic and pesticides either in drinking water or through vegetables, fruits, and other edible items with various concentrations above the WHO/FAO permissible limits. Being an agricultural country, a 1169% increase has been recorded with the use of different types of pesticides since last two decades, and almost similar rise in the burden of diabetes.

There is a growing global concern of arsenic and pesticides exposure with the incidence of DM. Besides other factors, the environmental attributors in the incidence of DM in Pakistan have not been conclusively elucidated yet which in turn deserve a resourceful targeted research.

## Introduction

Pollution is the addition of substances or energy to the environment, likely to produce harmful effects on human health and ecosystem. Heavy industrialization and scientific developments have led to the addition of detrimental chemicals to the environment in the form of heavy metals, agrochemicals, pesticides and hydrocarbons [1]. Environmental pollution has been a major health concern throughout the world since very long, but the risks are higher in underdeveloped countries. In low developed countries, environmental pollution contributes about 8-9% of total disease burden [2].

Pakistan, a country with a population exceeding 180 million, having four provinces along with federally administered tribal areas, is the 6th populous country of the world with a very small economy [3].

### Arsenic exposure in Pakistan

The presence of arsenic (As) in drinking water has become a major public health concern around the world. Arsenic has been recognized globally as the most toxic inorganic contaminant of drinking water [4]. Water sources of Asian countries, including Pakistan are among the most affected ones for As contamination [5,6].

### Regional status of arsenic exposure in Pakistan

#### Sindh province

Approximately 36% people in Sindh province consume drinking water or vegetables containing As above the WHO limit [7]. The ground water sources in some rural areas in Sindh province contain As up to 1.1 mg/L [8]. A study published by Arain et al. reported the contamination of vegetables with As. The vegetables grown in the south east part of Sindh contain As in the range of 0.90-120 mg/kg, exceeding WHO/FAO limit of 0.0001 mg/kg [9]. Drinking water samples tested from districts Tharparkar, Matiari and Jamshoro have been shown to contain As in the range of 0.013-2.09 mg/L [7,10,11]. Data about the presence of As in either drinking water or vegetable from other areas of Sindh are not available to completely understand the status of peoples in term of As exposure in this province.

#### Punjab province

Punjab is the most populated province of Pakistan. The drinking water sources of Punjab province have been reported to contain As level above the WHO safe limit. According to reports, approximately 20% population in Punjab is exposed to As contaminated water [12]. Drinking water sources of some regions in Punjab, such as Bahawalpur, Gujranwala, Kasur, Lahore, Multan, Muzaffargarh, Rahim Yar khan and Sheikhupura, have been reported to contain As level in the average range of 0.0794 to 0.9 mg/L [6,8,13-15]. “Pakistan Council of Research in Water Resources” a government body responsible for water quality in Pakistan, has reported As contents above the WHO permissible limits in drinking water sources of some other regions such as Attock, Multan, Sargodha and Bahawalpur. However, the values for As content in water samples of these areas have not been made public [16]. Overall, compared to other provinces of Pakistan, water samples of many areas of Punjab province have been studied for the presence of As contents. It has been reported that drinking water sources of Punjab province contain comparatively high As contents.

#### Khyber PakhtoonKhwa province

Arsenic from the soil gets its way to ground water and ultimately enters the crops. Beside presence of As in drinking water sources, it has been reported that wheat, which is the is the main edible crop of the Pakistani population, contain As contents above safe limits. Al-Othman et al. have studied wheat crop samples from different districts of Khyber PakhtoonKhwa for bioaccumulation of As contents. The results of this study showed that wheat crop in these areas contains As in the range of 0.005-1.113 mg/kg. The permissible limits of As in agronomic crops is 0.43 mg/kg [17]. However, WHO/FAO (Codex-1995 amended 2009) has not defined any permissible limit for As in the wheat crop Table 1.

**Table 1:** **Existence of As in drinking water sources of various regions of Pakistan**

**References**	**Sample studied**	**Arsenic**	**Result**
[15]	Drinking water	As	79.4 μg /L
[8]	Drinking water	As	1.5-5 μg /L
[14]	Drinking water	As	Reported as “above WHO limit”
[6]	Drinking water	As	10-906 μg/L
[5]	Drinking water	As	906 μg/L
[11]	Drinking water	As	3-106 μg/L
[16]	Drinking water	As	Reported as “above WHO limit”
[18]	Drinking water	As	32-1900 μg/L
[17]	Wheat crop	As	0.005-1.113 mg/kg
[9]	Vegetables	As	0.90-120 mg/kg

Maximum residue limit for As in the vegetable is 0.0001 mg/kg (WHO/FAO).

Maximum residue limit of As for wheat crop is 0.43 mg/kg (Al-othman et al. (2012).

The permissible limit for As in drinking water is 10 μg/L) (WHO 2006).

#### Balochistan province

Studies about the presence of As prevalence in Balochistan province are limited. However, some studies have reported the presence of As within the range of permissible limits of WHO in some selected districts [13].

#### Pesticides exposure in Pakistan

**Figure 1:**
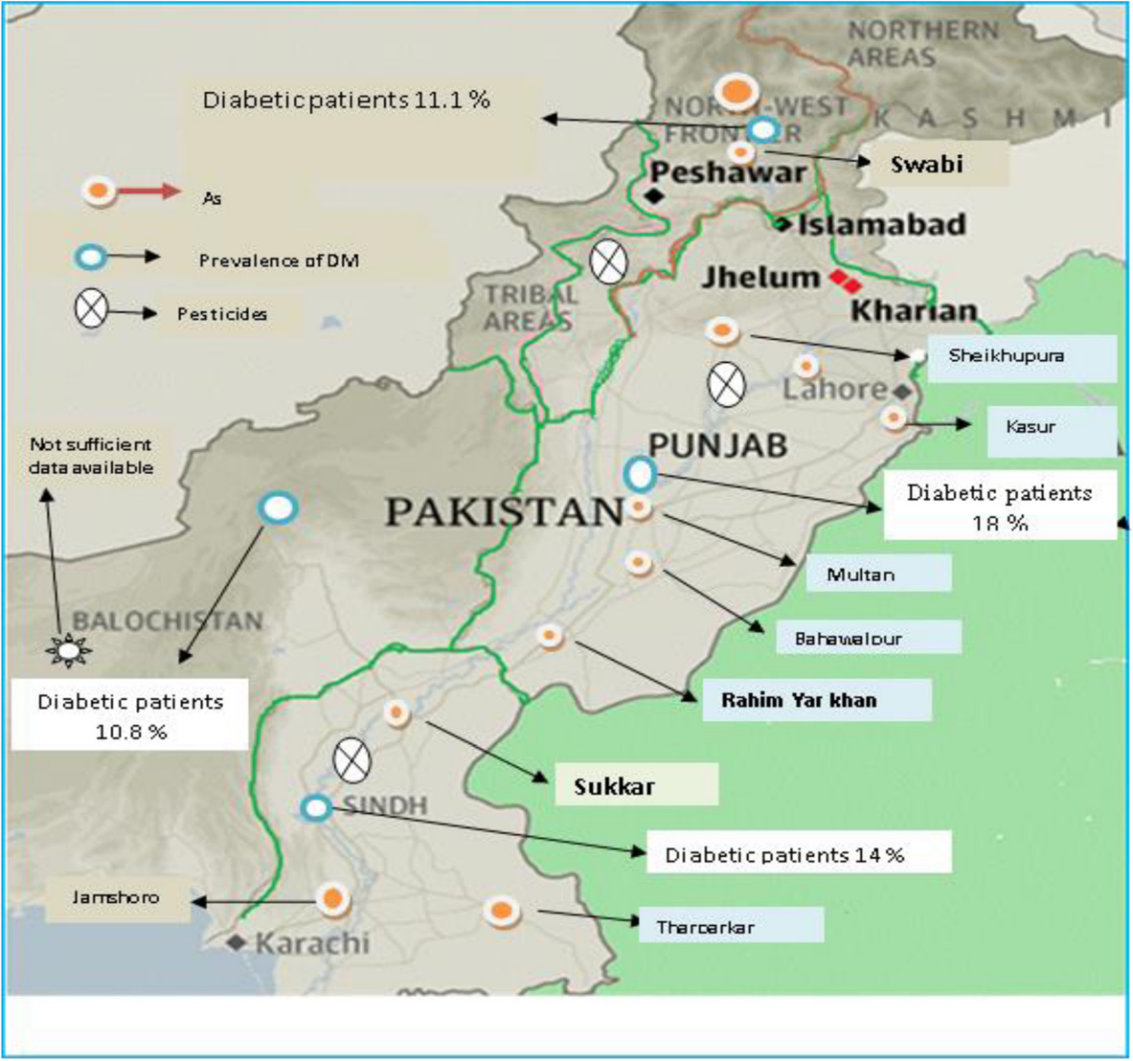
**Map representing areas with high contents of arsenic, pesticides and DM prevalence in Pakistan.**

Pesticides presence poses a great threat to the environment and human life. The use of pesticides in agriculture is substantially increasing from the last four decades for protection of crops [19,20]. It is astonishing to note that very small quantities of applied pesticides reaches the target organism accurately and major part of “applied pesticides” is dispersed in the environment and enters the human food chain [20]. Migration of applied pesticides in drinking water and subsequent entry to food chain has remained a global concern and several cases of drinking water contamination have been reported in the developed world [21]. Pakistan being an agriculture country consumes approximately 70 thousand tons of different pesticides annually and the use of different types of pesticides in Pakistan has increased by 1169% in the last 2 decades [22-24]. Each crop in Pakistan receives at least 10 different types of pesticides, which is an alarming signal for public health [23,25]. This huge application of pesticides in agriculture sector has led to the contamination of drinking water sources, vegetables, cattle food, milk, and fish samples throughout the country [26,27]. Some water sources of Punjab province have been reported by Tariq et al. to contain different types of pesticides like bifenthrin, cyhalothrin, carbofuran, endosulfan, methyl parathion and monocrotophos exceeding the permissible limits defined by WHO/FAO. Fruits and vegetable collected from various parts of the country and tested for the presences of different types of pesticides have been reported to contain pesticides like, carbofuran, dimethoate, deltamethrin, cypermethrin, and chlorpyrifos contents exceeding the WHO/FAO maximum residue limits [27-29]. Water sources of the cotton growing areas of, both Punjab and Sindh province, and tobacco growing areas of Khyber PakhtoonKhwa have been found contaminated with pesticides. As Pakistan is an agricultural country, therefore a huge amount of different pesticide use for protection of crops is imminent. There are published few studies regarding the contamination of vegetables and other food items with pesticide residues from all parts of the country. Determination of pesticides in vegetables and drinking water, has remained an understudied subject in Pakistan. All available studies about the prevalence of pesticides in various areas have been accumulated in Table 2, Figure 1.

**Table 2:** **Pesticides detected in water samples of various regions in Pakistan**

**Reference**	**Sample studied**	**Pesticides**	**(WHO/FAO) permissible limit**	**Result**
[24]	Ground water	Bifenthrin	ND	11 μg/L
		Carbofuran	7 μg/L	36 μg/L
		Methyl parathion	9 μg/L	3 μg/L
		Monocrotophos	3 μg/L epa	20 μg/L
		Carbofuran	7 μg/L	36 μg/L
		Endosulfan	ND	6 μg/L
		Cyhalothrin	ND	7 μg/L
[30,31]	Ground water	Dichlorvos	ND	0.03-0.45 μg/L
		Mevinphos	ND	0.06-0.21 μg/L
		Dimethoate	6 μg/L	0.0-0.15 μg/L
		Methyl– parathion	ND	0.0-0.06 μg/L
		Chlorpyrifos	30	0.0-0.03 μg/L
		Fenitrothion	ND	0.0-0.2 μg/L
		Endosulfan	ND	0.0-0.2 μg/L
		Profenphos	3 μg/L	0.01-0.17 μg/L
		Carbofuran	7 μg/L	0.0-0.26 μg/L
		Lindane	2 μg/L	0.11 μg/L
[27]	Fruits	Cypermethrin	0.1 mg/kg	0.94 mg/kg
		Deltamethrin	ND	0.039 mg/kg
		Dimethoate	ND	0.139 mg/kg
		Endosulfan	ND	0.774 mg/kg
[32]	Vegetables	Lindane	0.5 mg/kg	4.21 mg/kg
	Luffa	Cypermethrin	ND	1.63 mg/kg
		Methylparathion	ND	1.71 mg/kg
	Cauliflower	Methylparathion	0.2 mg/kg	2.5 mg/kg
		Methamidopos	1.0 mg/kg	2.60 mg/kg
		P,P, DDT	1.0 mg/kg	10.3 mg/kg
	Onion	Methylparathion	ND	3.15 mg/kg
		Methamidopos	0.5 mg/kg	4.61 mg/kg
		Cypermethrin	0.1 mg/kg	1.8 mg/kg
	Tomato	Malathion	3.0 mg/kg	10 mg/kg
		Fenvalerate	2.2 mg/kg	1.0 mg/kg

ND: not defined.

### Evidences for association of As and pesticides with incidence of diabetes

#### Arsenic

Enough experimental and epidemiological evidences are available which suggest that As exposure add to the incidence of diabetes [33-36]. Navas-Acien et al. have published a cross sectional study of 788 individuals chronically exposed to As in the USA. The researchers found a strong correlation between chronic exposure to low level of As and non-insulin diabetes mellitus [37]. Exposure to As, either in drinking water or from other environmental sources, and its possible link with incidence of diabetes has been reported in epidemiologic studies published from various parts of the world like Bangladesh, Taiwan, South Korea, Cyprus, Serbia, China and Mexico [35,38-43]. Recently a population based study published from Iran has suggested a relationship between even a lower exposure level of As (20–30 μg/L) and incidence of diabetes and hypertension [44]. Recently a workshop was conducted by the U.S. National Toxicology Program on environmental chemicals and the incidence of DM in which various epidemiological and experimental evidences were evaluated. It has been concluded that studies regarding As exposure with an incidence of DM are suggestive but not sufficient and further objective research in this area is highly recommended [45].

**Figure 2:**
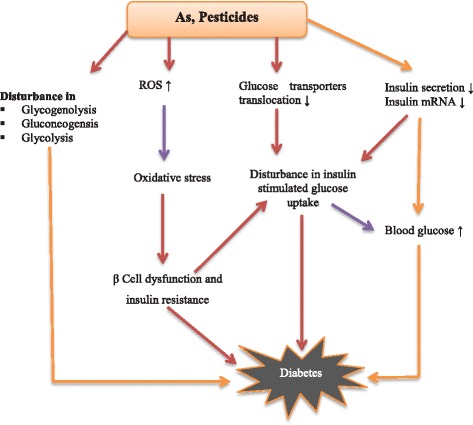
**A Schematic illustration of possible pathways by which As and pesticides may induce diabetes.**

Multiple mechanisms may be involved in As induced diabetes. In experimental studies As has been reported to act on multiple targets such as, affecting insulin sensitivity, altering β cells function, alteration of β cells signaling pathways, disturbing glucose production in the liver, and the reduction of insulin secretion and initiation of oxidative stress in the pancreas [46-49] Figure 2, Table 3. Evidences from population based and experimental studies mentioned above are suggestive enough to prove that chronic As exposure may lead to the incidence of diabetes.

**Table 3:** **Evidences for association of As and pesticides with diabetes**

**Reference**	**Type of study**	**Compound**	**Doses**	**Duration**	**Outcome/result**
[36]	Experimental	As	10 mg/kg	>3 months	Islets damage
					Insulin secretion ↓ reactive oxygen species (ROS) ↑
[50]	Experimental (Pancreatic β cells)	As	0.5-2 μM	>3 months	Insulin secretion ↓
					Insulin mRNA levels ↓
[49]	Experimental	As	5.55 mg/kg	30 days	Liver glycogen level ↓
					Blood sugar level ↑
					Glutamate pyruatetransaminsae activity ↓
					Glucose 6-phosphatase activity ↓
[48]	Experimental	As	1 mM	--	PI-3 kinase independent; SAPK2/p38
					IUF-1translocation from cytoplasm to nucleus ↑
[35]	Epidemiologic	As	-	>3 months	Positive association of As exposure with diabetes
[37]	Epidemiologic	As	-	>3 months	Positive association of As exposure with diabetes
[38]	Epidemiologic	As	-	>3 months	Positive association of As exposure with diabetes
[51]	Epidemiologic	As	-	>3 months	Positive association of As exposure with diabetes
[39]	Epidemiologic	As	-	>3 months	Positive association of As exposure with diabetes
[41]	Epidemiologic	As	-	>3 months	Positive association of As exposure with diabetes
[42]	Epidemiologic	As	-	>3 months	Positive association of As exposure with diabetes
[52]	Epidemiologic	As	-	>3 months	Positive association of As exposure with diabetes
[43]	Epidemiologic	As	-	>3 months	Positive association of As exposure with diabetes
[44]	Epidemiologic	As	-	>3 months	Positive association of As with diabetes in population
[53]	Experimental	Malation	100-400 ppm	30 days	Hepatic glycogen phosphorylase ↑
					Phosphoenol pyruvate carboxy kinase ↑
[54]	Experimental	Malation	25-100 mg/kg/day	32 days	Phosphoenol pyruvate carboxy kinase
					Glucose 6-phosphatase ↑
[55]	Epidemiologic	Organo -chlorine Pesticides	-	>3 months	Positive association of pesticides with diabetes Insulin resistance observed
[56]	Epidemiologic	Organo -chlorine Pesticides	-	>3 months	Positive association of pesticides with diabetes
[57]	Epidemiologic	Malation	-	>3 months	Positive association of pesticides with diabetes
					Insulin resistance observed

#### Pesticides

Numerous experimental studies are available about the toxic effects of pesticides and the occurrence of type 2 diabetes. Organophosphorus and organochlorine types of pesticides in particular have been mentioned to possess deleterious effect on glucose metabolism and insulin secretion [53,55,57]. However, few epidemiologic studies exist in the literature about the role of pesticides in the occurrence of diabetes [58,59]. A prospective study published by Montgomery et al. have reported a high prevalence of diabetes in the 33457 licensed pesticide applicators in the US, and the ratio of diabetes incidence was noted more in organochlorine and organophosphorus type of pesticide applicators [56]. Pesticides may cause diabetes by affecting multiple pathways involved in glucose regulation. The involved mechanisms include oxidative stress, nitrosative stress, pancreatitis, inhibition of choline esterase, altered mitochondrial functions, and alteration of adrenal gland functions [54,60-62] (Figure 2, Table 3).

#### Overview of DM prevalence in Pakistan

**Figure 3:**
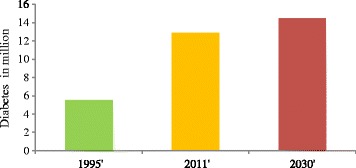
**Trend in the rise of diabetic patients in Pakistan.**

**Figure 4:**
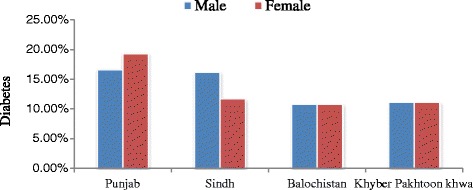
**Regional and gender wise prevalence of diabetes in Pakistan.**

DM is the leading chronic diseases and has emerged a big socioeconomic burden in Pakistan. Various populations-based studies and national surveys have shown that DM is a highly prevalent disease in almost all regions of Pakistan, with an overall ratio of 22.04% in urban and 17.15% in rural areas [63-65]. According to various surveys, the pattern of DM prevalence in Pakistan is as Punjab; male, 16.6%, female, 19.3%, Khyber PakhtoonKhwa; 11.1% both sexes, Balochistan; 10.8% both sexes, Sindh; male 16.2% female 11.7% [66-68]. According to the latest data provided by the International Diabetes Federation, (www.Idf.Org/Diabetesatlas/Data-Visualisations) Pakistan is among the leading countries with high prevalence of DM. As per WHO 2011 report total prevalence of diabetes is 12.9 million among them 9.4 million are diagnosed, while 38 million population is pre-diabetic and the number of diabetic patients would increase to 14 million by 2030 [69] Figures 3 and 4.

## Discussion and conclusion

Like other poor countries, polluted water is also one of the major public health threats in Pakistan with special emphasis to drinking water. Pakistan stands at position 80 in the world community in term of drinking water quality. Water used for drinking purposes is contaminated with heavy metals and pesticides [22]. About 89% of drinking water sources have poor quality and are not according to the parameters set by WHO for human consumption [14,18]. Pakistan is an agricultural country, and for the sake of high crop yield, there has been recorded about 1169% increase in the use of different types of pesticides for the last two decades, and almost similar rise in the burden of diabetes, and according to various studies the number of diabetic patients in Pakistan would become two fold by 2030. According to World Bank Report 2010, Pakistan is facing health crises with high incidence of diabetes, affecting poor families which increase the burden of poverty (Figure 3).

Experimental and some epidemiologic studies gathered above suggest that environmental pollutants such as pesticides, As, Cd and Hg are risk factors in the incidence of DM. Excessive As presence has been reported in water sources of many other countries of the World like Argentina (1–9900 μg/L), China (0.05-850 μg/L), India (10–3200 μg/L), Brazil (0.5-350 μg/L), Mexico (8–620 μg/L), USA (1–100,000 μg/L), Taiwan (10–1820 μg/L), and particularly Bangladesh having 1–2500 μg/L [70-72]. And according published data As exposure has been considered as one of the risk factors in the incidence of DM in these countries and some countries like China, India, Brazil and USA are at the forefront of this epidemic (http://www.idf.org/diabetesatlas/data-visualisations).

We are of the opinion that there might be a correlation of heavy metals like As and pesticide exposure with the prevalence of DM in Pakistan. Even though, there are many regions, where the drinking water sources are grossly contaminated with heavy metals and pesticides, but to correlate the prevalence of DM in exposed population and exposure to these pollutants; there is a complete lack of targeted epidemiologic research in the affected areas. We are of the view that along with other confounding factors, the role of As and pesticides in the epidemic of DM in Pakistan cannot be overlooked. Furthermore, there is a need of an urgent attention of concerned quarters like environmental researchers/toxicologists, governmental and non-governmental organizations to objectively evaluate the epidemics of DM in Pakistan from various dimensions including identifying the role of environmental pollutants and to take practical steps in this regard. No doubt, comprehensive and objective data derived from a resourceful targeted research on environmental pollutants and the prevalence of DM in Pakistan would be extremely laudable among environmental scientists. Data obtained from such targeted research will significantly add to the growing body of evidences about the role of environmental pollutants in the occurrence of chronic diseases.
